# Demoralization in oral cancer inpatients and its association with spiritual needs, quality of life, and suicidal ideation: a cross-sectional study

**DOI:** 10.1186/s12955-022-01962-6

**Published:** 2022-04-02

**Authors:** Ting-Gang Chang, Pei-Ching Huang, Chiann-Yi Hsu, Ting-Ting Yen

**Affiliations:** 1 Department of Psychiatry, Taichung Veterans General Hospital, Taichung, Taiwan; 2 School of Psychology, Chung Shan Medical University, Taichung, Taiwan; 3 Cancer Prevention and Control Center, Taichung Veterans General Hospital, Taichung, Taiwan; 4 Biostatistics Task Force of Taichung Veterans General Hospital, Taichung, Taiwan; 5 Department of Otorhinolaryngology, Taichung Veterans General Hospital, 1650 Taiwan Boulevard Sect. 4, Taichung, 40705 Taiwan, ROC; 6 School of Medicine, National Yang Ming Chiao Tung University, Taipei, Taiwan

**Keywords:** Demoralization, Oral cancer, Spirituality, Quality of life, Suicidal ideation

## Abstract

**Background:**

Demoralization is a common problem in oral cancer patients owing to the chronic and severe nature of their affliction. However, the association between demoralization and the patient’s spiritual needs, quality of life, and suicidal ideation remains unclear. This study aims to provide insights into possible links between demoralization among oral cancer patients and its effects on the patient’s spiritual needs, quality of life, and suicidal ideation.

**Methods:**

We examined 155 Taiwanese oral cancer inpatients in Taichung Veterans General Hospital, Taiwan, using the following three rating scales: (a) Demoralization Scale Mandarin Version (DS-MV), (b) Spiritual Interests Related to Illness Tool, and (c) The Taiwan Chinese versions of the European Organization for Research and Treatment of Cancer Quality of Life Questionnaire. Suicidal ideation was established if at least one of the two suicide-related items on the DS-MV scale were checked. We divided the participants into high- and low-demoralization groups, per the cutoff score of 30. We then explored group associations with sociodemographic features, quality of life, and spiritual needs. Logistic regression and receiver operating characteristic (ROC) curves were used to determine demoralization and its association between these variables.

**Results:**

Fifty-five (35.5%) patients were categorized as having high demoralization (DS-MV scale score > 30), with scores for DS-MV for all patients being 27.2 ± 16.8. The rates of suicidal ideation were 29.1% (16/55) in the high-demoralization group and 2% (2/100) in the low-demoralization group, with an odds ratio (95% confidence interval) of 20.10 (4.41–91.55). Logistic regression analysis revealed significant effects of spiritual needs and global health status on the DS-MV scores (*p* < 0.001). Multivariate analyses further confirmed that only overall quality of life scores < 62.5 and spiritual needs < 3.7 significantly predicted the occurrence of high demoralization.

**Conclusion:**

High demoralization is associated with low satisfaction with spiritual needs, poor quality of life, and high risk of suicidal ideation. DS-MV may potentially be an effective tool for achieving holistic health care among oral cancer patients.

## Background

Oral cancer is the sixth most prevalent form of cancer in Taiwan. It is especially common among Taiwanese men in the 30–50-year age group. Currently, surgical excision with or without adjuvant therapy is the accepted treatment modality [1]. However, there has been an increase in the need for post-surgical medical care among oral cancer patients. Patients with oral cancer experience uncomfortable post-surgical outcomes including distorted appearance, poor articulation, difficulty in mastication, uneasy respiration post-tracheostomy, and exposed surgical wounds along with post-surgical inflammation. Quality of life is an important outcome in oral cancer [2]. Quality of life of patients with oral cancer is affected by a number of factors, such as age, sex, site, stage, emotional status, smoking and alcohol consumption, marital status, income, performance status, method of reconstruction, access, mandibular resection, neck dissection, percutaneous endoscopic gastrostomy, and postoperative radiotherapy [3]. Patients of head and neck cancer face a number of survivorship pitfalls post-surgery, like sexual dysfunction and negative body image, psychosocial issues, including depression, anxiety, and suicide risk [4–6]. Psychological issues, such as depression and anxiety, may persist even after the physical symptoms have been stabilized [7].

Demoralization is a normal psychological response to painful, advanced, and/or terminal diseases [8, 9] and currently does not refer to specific brain pathologies. In contrast to major depression, demoralization usually manifests as existential distress, helplessness, hopelessness, and the loss of meaning and purpose associated with a specific event [10]. Demoralization exists independently of depression among patients with chronic and/or severe illnesses [11]. Demoralization is of high clinical importance when symptoms are severe or serious suicidal thoughts occur.

A review study reported demoralization in 13–18% patients with progressive disease or cancer [12]. Some evidence supports a relationship between demoralization and sociodemographic factors, such as unemployment, mental problems, or a lack of spiritual needs being met as well as other psychological factors, such as distress and a lack of hope, goals, and value for life [9]. In Taiwan, 24.3% of patients with head and neck cancers experience demoralization, with high Demoralization Scale Mandarin Version (DS-MV) scores, particularly those with no jobs or low incomes [13]. Other studies have reported an association between demoralization and poor quality of life [14–16]. However, the prevalence of demoralization in patients with oral cancer remains unclear. Clinicians and psychiatrists are more familiar with the diagnosis of depression than with the definition, diagnosis, evaluation, or intervention of demoralization.

Poor quality of life may also be an important determinant that propels terminal cancer patients to seek accelerated death [17]. Demoralization has a greater impact on suicidal ideation than on depression among patients with lung cancer, leukemia, and lymphoma; demoralization also has a greater impact on suicidal ideation than depression [18]. Reportedly, 0.8–71.4% of non-psychiatric cancer patients experience suicidal ideation, with severity likely related to the site of cancer, physical functioning, and prognosis [19]. Baker et al. [20] first showed that hopelessness is an independent mediator of suicide and a predictor of suicidal tendencies more effectively than depression [21]. Demoralization may be an indicator of suicidal ideation in cancer patients who are not depressed and have already lost their meaning of life [18]. Depression, loss of meaning and purpose, loss of control, and low self-worth are strong clinical indicators of suicidal tendencies [17]. Therefore, existentially oriented interventions, such as meaning-centered therapy, are needed for early suicide prevention and treatment.

Patients with cancer who meet their spiritual needs are better able to cope with the physical and mental distress and consequences of cancer treatment. For cancer patients, spiritual needs strongly affect their quality of life [22]. Spiritual needs are multidimensional and are related to an individual’s religious and cultural background. Spiritual needs can be constructed along four dimensions: existential needs, religious needs, inner peace needs, giving/generativity, and forgiveness needs [23]. The spiritual needs of patients are extensive and personal [24]. Past research on cancer patients has shown that satisfaction with one’s spiritual needs is positively related to quality of life [25]. To achieve holistic care, it is imperative to cater to the spiritual aspects of patients in addition to treating them within the bio-psycho-social treatment model. There are no data on the link between demoralization and the spiritual needs and quality of life in patients with oral cancer.

Therefore, we aimed to understand severity and incidence of demoralization in oral cancer patients and its association between demoralization and spiritual needs, quality of life, and suicidal ideation in oral cancer patients. We also try to predict the demoralization in oral cancer patients in order to provide timely diagnosis and interventions. Our results will also serve to further improve the quality of life and overall care quality of patients with oral cancer.

## Methods

### Design

This cross-sectional study aimed to examine the relationship between demoralization and spiritual needs, quality of life, and suicidal ideation among inpatients diagnosed with oral cancer.

### Participants

Convenience sampling was used to recruit the sample. Oral cancer patients hospitalized in acute care at Taichung Veterans General Hospital, Taichung, Taiwan, for various reasons from August 2018 to August 2019 were invited to participate in this study. The inclusion criteria were diagnosis of oral cancer, aged ≥ 20 years, and ability to communicate in Mandarin. The exclusion criteria were an inability to communicate in Mandarin or complete the questionnaires.

### Instruments

Our trained research assistants conducted surveys on DS-MV [13] and appropriate sociodemographic variables (age, sex, education, marital status, religious affiliation, employment status, monthly income), cancer status (stage and location), treatment, quality of life, sleep quality, spiritual needs scale, duration since diagnosis, Spiritual Interests related to Illness Tool (C-SpIRIT), and The Taiwan Chinese versions of the European Organization for Research and Treatment of Cancer Quality of Life Questionnaire (EORTC QLQ-C30).

#### Demoralization

DS-MV was translated from the Demoralization Scale developed by Kissane [26]. The DS-MV has the following dimensions: loss of meaning (5 items), disheartenment (6 items), dysphoria (5 items), sense of failure (4 items), and helplessness (4 items). The DS-MV has acceptable reliability with a Cronbach’s alpha of 0.928 and internal reliability is acceptable with Cronbach’s alphas in the range of 0.63–0.85. Pearson’s correlation showed a positive correlation (γ = 0.703, p < 0.001) between DS-MV and the Beck Hopelessness Scale scores and a negative correlation (γ =  − 0.680, p < 0.001) between DS-MV and the McGill Quality of Life Questionnaire scores. According to previous reports, DS-MV scores > 30 indicated high demoralization [26, 27].

#### Suicidal ideation

There are two items from the DS-MV that indicate suicidal ideation: “life is no longer worth living” and “I would rather not be alive.” Suicidal ideation was defined as answering “yes” to at least one of these statements.

#### Questionnaire on Quality of life

Our questionnaire was derived from EORTC QLQ-C30, developed by the EORTC QoL Group. It has been translated and validated in over 100 languages and is used in numerous studies worldwide. The Chinese version of the EORTC QLQ‐C30 questionnaire in Taiwan was obtained with an agreement license from the Quality of Life Unit of the EORTC Data Centre in Brussels, Belgium. The questionnaire contained items designed to evaluate general health, along with physical, emotional, and social domains. It includes 30 questions grouped into five functional scales: physical, role, cognitive, emotional, and social. It has three symptom scales: fatigue, nausea, and pain, as well as six single questions evaluating the intensity of the following symptoms: dyspnea, sleeplessness, lack of appetite, constipation, diarrhea, and financial problems. The last two questions revealed the overall health assessment. The questionnaire contains a 4-point scale (never: 1, sometimes: 2, often: 3, very often: 4) [28]. A high score indicated a high response level. A high global health status represents high quality of life, and a high symptom score represents a high level of symptoms.

#### Spiritual Interests Related to Illness Tool (C-SpIRIT) Chinese version

Taylor developed a 44-item SpIRIT to measure satisfaction with the spiritual needs of cancer patients and their families [29]. The eight dimensions of the 44-item SpIRIT were as follows: possessing a positive perspective, having a relationship with God, giving love to others, receiving love from others, revaluating beliefs, seeking the meaning of life, practicing religion, and preparing for death. A 5-point Likert scale was used for responding. The higher the score, the higher the spiritual need of a specific category. Lin [30] modified the Chinese version of the Spiritual Needs Measurement Tool for Taiwanese patients. There are 21-items divided into five dimensions related to beliefs/religion, positive attitude toward life, love to/from others, seeking the meaning of life, and a peaceful mind.

### Data collection

We divided the participants into two groups: high- and low-demoralization groups, per the cutoff score of 30. We then explored group associations with sociodemographic features, quality of life, and spiritual needs. Logistic regression and receiver operating characteristic (ROC) curves were used to determine the demoralization and its association between overall quality of life, spiritual needs.

### Statistical analyses

Sociodemographic and cancer associated data are expressed as numbers and percentages. Between-group differences were evaluated using the chi-square test, Fisher’s exact test, the Mann–Whitney test, logistic regression, and Spearman’s rank correlation coefficient. Statistical significance was set at p < 0.05. Analyses were performed using the Statistical Package for the Social Sciences (IBM SPSS version 22.0; International Business Machines Corp, New York, USA).

### Ethical statement

Informed consent was obtained from all participants. This study was approved by the Ethics Committee of the Taichung Veterans General Hospital (serial number: CE18244B). The researchers explained the voluntary participation, guarantee of anonymity, and freedom to withdraw from the study at any time.

## Results

We recruited 155 inpatients with oral cancer for the study. The sociodemographic characteristics and medical information are presented in Table 1. Those who had completely filled out the DS-MV, C-SpIRIT, EORTC QLQ-C30 questionnaires included 147 men (94.8%) and 8 women (5.2%) with a mean age of 52.9 ± 9.7 years. Among them, 76.8% were married, 54.2% had a high school degree or above, 53.5% were religiously affiliated with Taoism, 67.1% had full-time jobs, 53.5% were diagnosed with stage III/IV cancer, and 85.2% were admitted for surgery.

**Table 1:** Sociodemographic characteristics of all patients and associations between the variables for the two groups

	Total	DS-MV˂ = 30 (n = 100)	DS-MV > 30 (n = 55)	*p* value
Age	52.9 (45.4–59.2)	53.3 (44.8–59.8)	52.2 (47–59.2)	0.982
Gender				0.455
Male	147 (94.8%)	96 (96.0%)	51 (92.7%)	
Female	8 (5.2%)	4 (4.0%)	4 (7.3%)	
Education				0.627
Elementary school	16 (10.3%)	10 (10.0%)	6 (10.9%)	
Junior school	55 (35.5%)	35 (35.0%)	20 (36.4%)	
Senior high school	68 (43.9%)	47 (47.0%)	21 (38.2%)	
College	13 (8.4%)	6 (6.0%)	7 (12.7%)	
Research institute	3 (1.9%)	2 (2.0%)	1 (1.8%)	
Marital status				0.743
Married	119 (76.8%)	79 (79.0%)	40 (72.7%)	
Never married	23 (14.8%)	14 (14.0%)	9 (16.4%)	
Divorced	10 (6.5%)	5 (5.0%)	5 (9.1%)	(9.1%)
Widowed	3 (1.9%)	2 (2.0%)	1 (1.8%)	
Religious affiliation				0.261
Atheist	21 (13.5%)	14 (14.0%)	7 (12.7%)	
Taoism	83 (53.5%)	58 (58.0%)	25 (45.5%)	
Buddhism	40 (25.8%)	21 (21.0%)	19 (34.5%)	
Christianity	3 (1.9%)	1 (1.0%)	2 (3.6%)	
Other	8 (5.2%)	6 (6.0%)	2 (3.6%)	
Employment status				0.394
Full-time job	104 (67.1%)	69 (69.0%)	35 (63.6%)	
Retired	22 (14.2%)	14 (14.0%)	8 (14.5%)	
Part-time job	17 (11.0%)	8 (8.0%)	9 (16.4%)	
Unemployment	9 (5.8%)	6 (6.0%)	3 (5.5%)	
Housewife/househusband	3 (1.9%)	3 (3.0%)	0 (0.0%)	
Monthly income(NTD)				0.069
$20,000 or less	36 (23.2%)	21 (21.0%)	15 (27.3%)	
$20,001–$40,000	59 (38.1%)	39 (39.0%)	20 (36.4%)	
$40,001–$60,000	41 (26.5%)	23 (23.0%)	18 (32.7%)	
$60,001 or more	19 (12.3%)	17 (17.0%)	2 (3.6%)	
Tumor stage				0.567
0	2 (1.3%)	2 (2.0%)	0 (0.0%)	
I	31 (20.0%)	20 (20.0%)	11 (20.0%)	
II	39 (25.2%)	23 (23.0%)	16 (29.1%)	
III	21 (13.5%)	16 (16.0%)	5 (9.1%)	
IV	62 (40.0%)	39 (39.0%)	23 (41.8%)	
Treatment				0.303
Surgery	132 (85.2%)	88 (88.0%)	44 (80.0%)	
Chemotherapy	10 (6.5%)	6 (6.0%)	4 (7.3%)	
Symptom relief	12 (7.7%)	5 (5.0%)	7 (12.7%)	
Immunotherapy	1 (0.6%)	1 (1.0%)	0 (0.0%)	
Time since diagnosis				0.185
1–2 years	11 (7.1%)	6 (6.0%)	5 (9.1%)	
< 3 months	83 (53.5%)	60 (60.0%)	23 (41.8%)	
3 months–1 year	17 (11.0%)	10 (10.0%)	7 (12.7%)	
> 2 years	44 (28.4%)	24 (24.0%)	20 (36.4%)	
Recurrence (Yes)	41 (26.5%)	22 (22.0%)	19 (34.5%)	0.133
Under anti-cancer treatment(Yes)	145 (100%)	96 (96.0%)	49 (89.1%)	0.168
Location				0.083
Tongue	61 (39.4%)	37 (37.0%)	24 (43.6%)	
Buccal	44 (28.4%)	35 (35.0%)	9 (16.4%)	
Gum	26 (16.8%)	16 (16.0%)	10 (18.2%)	
Mouth	19 (12.3%)	11 (11.0%)	8 (14.5%)	
Lip	4 (2.6%)	1 (1.0%)	3 (5.5%)	
Oropharynx	1 (0.6%)	0 (0.0%)	1 (1.8%)	
Suicidal ideation(Yes)	18 (11.6%)	16 29.1%	2 2%	< 0.001*
Global health status/QoL	50.0 (33.3-66.7)	58.0 (41.8–75)	50.0 (17–58.3)	< 0.001*
Functional scales	69.0 (57-81)	77.2 (63.3–86.6)	59.4 (46.3–69.3)	< 0.001*
Physical functioning	73.3 (60-87)	80.0 (61.7–93)	67.0 (46.7–86.7)	0.015
Role functioning	66.7 (33.3-100)	66.7 (33.3–100)	50.0 (33–100)	0.048
Emotional functioning	75.0 (66.7-91.7)	83.3 (67–100)	66.7 (50–75)	< 0.001*
Cognitive functioning	83.3 (66.7-100)	83.3 (67–100)	66.7 (50–83.3)	< 0.001*
Social functioning	66.7 (33.3-83.3)	66.7 (50–83.3)	33.3 (33–66.7)	< 0.001*
Symptom scales	25.9 (16-35.8)	22.5 (12.7–31.3)	34.6 (24.1–41.4)	< 0.001*
Fatigue	33.3 (22.2-55.6)	33.3 (11.1–44.4)	44.4 (33.3–66.7)	< 0.001*
Nausea and vomiting	0.0 (0-16.7)	0.0 (0–16.7)	0.0 (0–16.7)	0.097
Pain	33.3 (16.7-66.7)	33.0 (16.7–50)	50.0 (33.3–66.7)	< 0.001*
Dyspnoea	0.0 (0-33.3)	0.0 (0–33.3)	33.0 (0–33.3)	0.033
Insomnia	33.3 (33-66.7)	33.3 (0–66.7)	33.3 (33–66.7)	0.319
Appetite loss	0.0 (0-33.3)	0.0 (0–33.3)	33.0 (0–33.3)	0.096
Constipation	0.0 (0-33.3)	0.0 (0–33.3)	33.3 (0–33.3)	< 0.001*
Diarrhoea	0.0 (0-33.3)	0.0 (0–33.3)	0.0 (0–33.3)	0.591
Financial difficulties	33.3 (0-66.7)	33.0 (0–33.3)	33.3 (33–66.7)	< 0.001*
Spiritual interests	3.8 (3.5-4.2)	4.0 (3.7–4.4)	3.6 (3.2–3.9)	< 0.001*
Related to beliefs	3.0 (2.6-3.6)	3.0 (2.6–3.6)	2.9 (2.4–3.3)	0.055
Religion, positive attitudes toward life	4.0 (3.8-4.8)	4.3 (4–5)	3.8 (3–4)	< 0.001*
Love to/from others	4.0 (3.8-4.5)	4.0 (3.8–4.5)	3.8 (3.5–4)	0.003*
Seeking for the meaning of life	4.0 (3.7-4.3)	4.0 (4–4.7)	4.0 (3–4)	0.001*
Peaceful mind	4.0 (3.7-4.7)	4.3 (4–5)	4.0 (3.3–4.3)	< 0.001*

Chi-square test. Fisher's exact test. Mann–Whitney U test. *p < 0.01

Continuous data were expressed median and IQR

Categorical data were expressed number and percentage

The mean total DS-MV score was 27.2 (± 16.8; range 0–70). Overall, 55 had high DS-MV scores, with a 35.5% incidence of demoralization, and 100 patients (64.5%) had low DS-MV scores. Sociodemographic features, cancer status, treatment, quality of life, sleep quality, spiritual needs scale, and duration since diagnosis are shown in Table 1 for all participants and the two groups (high and low DS-MV scores). We found no intergroup differences in sociodemographic features, cancer status, treatment duration since diagnosis, or recurrence status.

Regarding quality of life, all subscales of the functional scales had significantly lower scores in the high DS-MV group. In the same group, only fatigue, pain, dyspnea, constipation, and financial difficulties showed significantly higher scores. Regarding spiritual needs, a positive attitude toward life, love shown to/from others, seeking the meaning of life, and having a peaceful mind were associated with significantly low scores.

Eighteen patients (11.61%) agreed to at least one of the suicidal ideation statements. The suicidal ideation rates in the high- and low-demoralization groups were 16/55 (29.1%) and 2/100 (2%), respectively, with a significant intergroup difference (p < 0.001). The high-demoralization group was 20.1 times more likely to have suicidal ideation than the low-demoralization group, with an odds ratio (95% confidence interval) of 20.10 (4.41–91.55).

**Fig. 1:**
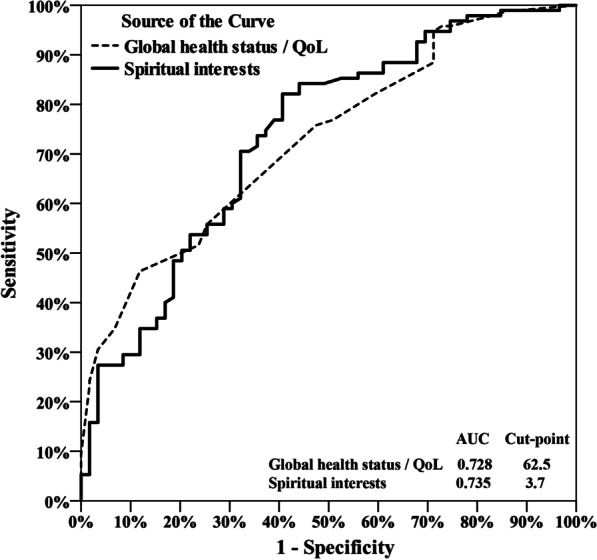
Receiver operating characteristic curve analysis of global health status/health quality and spiritual interests for demoralization

Logistic regression and ROC curves were used to determine correlations between overall quality of life, spiritual needs, and each patient’s demoralization scores. Results from univariate analyses showed that to predict the occurrence of high demoralization (AUC > 0.7), the following conditions were required: an overall quality of life score < 62.5, a functional score < 68.2, an asymptomatic score > 26.2, and a total score of spiritual needs < 3.7. Multivariate analyses further showed that only an overall quality of life total score < 62.5 and a spiritual needs score < 3.7 significantly predicted the occurrence of high demoralization (Table 2; Fig. 1).

**Table 2:** Univariate and multivariate analysis of demoralization in all patients

	Univariate			Multivariate		
	OR	95%CI	*p* value	OR	95%CI	*p* value
Global health status/QoL (< 62.5 vs. ≥ 62.5)	5.39	(2.22–13.07)	< 0.001*	5.09	(2.00–12.94)	0.001*
Functional scales (< 68.2 vs ≥ 68.2)	5.68	(2.73–11.85)	< 0.001*			
Symptom scales (≥ 26.2 vs. < 26.2)	4.15	(2.04–8.44)	< 0.001*			
Spiritual interests (< 3.7 vs. ≥ 3.7)	5.32	(2.59–10.90)	< 0.001*	5.08	(2.38–10.81)	< 0.001*

Logistic regression. *p < 0.01

## Discussion

This is the first study on patients with oral cancer regarding the associations between demoralization and spiritual needs, quality of life, and suicidal ideation. We found that (a) patients with oral cancer were more likely to experience demoralization (35.5%) than those with other cancers, (b) high demoralization was associated with low satisfaction in spiritual needs, (c) high demoralization was associated with low quality of life, and (d) the odds ratio of suicidal ideation was 20 times more for patients with higher demoralization than for those with lower demoralization. Therefore, we propose that early assessment of demoralization is helpful in achieving holistic healthcare and preventing early suicide in these patients.

This study found that cancer status, treatment duration, and recurrence status were not significantly associated with demoralization. Clinically, major surgery has a negative impact not only on demoralization rates, but also on the quality of life, satisfaction of spiritual needs, and suicidal ideation. The participants in the present study comprised a higher proportion of those with demoralization than those in a previous study [12]. However, the mean demoralization scores in the present study were lower than those in a previous study among outpatients with head and neck cancer in Taiwan [13]. A possible reason for this is that most participants were in the advanced stage. They were admitted for major surgery and underwent assessment before surgery.

Suicide is an important clinical issue in chronic cancer care, and our study evaluated suicidal ideation using the DS-MV. In our study, we used two DS-MV statements to define suicidal ideation: “Life is no longer worth living” and “I would rather not be alive.” Demoralization was positively associated with suicidal ideation. Our results showed that the highly demoralized group experienced greater suicidal ideation. This finding is consistent with the literature on patients with cancer [13, 18]. For patients with high demoralization, suicidal ideation is suspected; therefore, early intervention is needed. Furthermore, demoralization is more likely to associate with depression [8, 31]. Therefore, patients with comorbidities of depression and hopelessness experience greater demoralization and require early suicide prevention and treatment.

In this study, we did not distinguish between depression and demoralization. Demoralization and depression have different clinical manifestations and treatments. Demoralization is associated with poorly controlled physical symptoms, inadequately treated depression and anxiety, reduced social functioning, unemployment, marital status [12], and, possibly, age and sex [9]. With regard to these parameters, we found no difference between the high- and low-demoralization groups in the relevant factors, including age, sex, education, marital status, religious affiliation, employment status, monthly income, cancer status (stage and location), and treatment. The roles of these factors, including the differences between inpatient and outpatient standing, cancer stage, and other complications, are still not fully understood. Follow-up research needs to include prospective research and establishment of important treatment time points as well as physical, psychological, and spiritual needs of the patients, and resources available for long-term care.

Currently, several scales are used to identify demoralized patients, including the Demoralization Scale [26], DS-II [9], and the Short Demoralization Scale (SDS) [32]. We used the Mandarin version of the Demoralization Scale; there is currently no Mandarin version of the DS-II or SDS for evaluating demoralization. Medical professionals have poor understanding of demoralization and require sensitization and training [33]. DS-MV is a self-report questionnaire for oral cancer patients and should be used as a routine screening tool to predict quality of life, suicidal ideation, comorbid depression, and spiritual needs. For those facing survival threats, demoralization is a clinically useful feature that guides clinicians in their efforts to restore morale, meaning, and purpose [9].

Another key point of this study was to evaluate which factors could predict high levels of demoralization in patients with cancer. The results of the C-SpIRIT and EORTC QLQ-C30, an overall quality of life score < 62.5, a functional score < 68.2, an asymptomatic score > 26.2, and a total score of spiritual needs < 3.7 were all predictors of high demoralization. Clinically, the C-SpIRIT and EORTC QLQ-C30 can be used to infer high demoralization in patients.

Our study revealed the values of 27.2 ± 16.8 for DS-MV and a 35.5% incidence of demoralization in our patients. Oral cancer is usually included among head and neck cancers. A study reported that among patients with head and neck cancer, 50% reported problems with eating, 28.5% had depressive symptoms, and 17.3% experienced substantial pain [5]. In a previous study in Taiwan, head and neck cancer outpatients had the highest score on DS-MV (38.4, SE = 13.6) when compared with other cancer outpatients [13]. A previous study reported that patients with head and neck cancer comprised higher proportions of those with depression and anxiety [34]. Patients likely have greater demoralization when specific attributes of oral cancer, i.e., disease severity and postoperative treatment, are considered, as verified by our results. High demoralization is significantly correlated with subscale scores of quality of life (fatigue, pain, dyspnea, constipation, and financial difficulties) as well as subscale scores of spiritual needs (positive attitude toward life, love shown to/from others, seeking the meaning of life, and a peaceful mind). In our study, all participants were inpatients, with most being men with full-time jobs and religious affiliations, and had a minimum education level of senior high school. The results may vary depending on country, culture, disease characteristics, and general conditions.

### Limitations

This was a cross-sectional investigation based on patients from a single institute in Taiwan in which most oral cancer patients were men; therefore, the results are applicable mostly to male patients and the effects of sex remain unclear. Further, the reliability and validity of the DS-MV scale in identifying suicidal ideation have not yet been established. Therefore, suicidal ideation was the only descriptive statistical result.

A considerable amount of time is required to assess the overall needs of such patients and that the DS-MV is correlated with C-SpIRIT and EORTC QLQ-C3. Thus, high demoralization is negatively associated with low satisfaction with one’s spiritual needs as well as poor quality of life and high suicidal ideation. DS-MV may serve as an integrated assessment tool to help better understand the overall psychological care needs of these patients.

## Conclusions

We have shown that demoralization negatively correlated with quality of life satisfaction and spiritual needs in patients with oral cancer. Spiritual needs and quality of life scales predict demoralization. Demoralization is an important aspect to consider for holistic care in patients with oral cancer; therefore, further prospective studies are needed.

## Data Availability

All data generated in this study is included in this published article.

## Abbreviations

DS-MV: Demoralization Scale Mandarin Version
C-SpIRIT: Spiritual Interests Related to Illness Tool
EORTC QLQ-C30: The Taiwan Chinese versions of the European Organization for Research and Treatment of Cancer QoL Questionnaire
